# Preparation of Cavities

**Published:** 1874-04

**Authors:** C. E. Francis

**Affiliations:** New York.


### ARTICLE VII.

### *Preparation of Cavities.*

BY C. E. FRANCIS, D. D. S., NEW YORK.

The importance of properly preparing cavities in teeth to be filled is a matter apparently too little considered by many of our fraternity, and especially is this the case with some of the younger members of the profession. Not a few operations prove failures from lack of thoroughness in this respect. Fillings become undermined with decay; the margins of cavities crumble, or walls break entirely away; fillings loosen and a destruction of crowns ensues.

Even where fillings are well impacted, with evidences of good intent on the part of the operator, at an expenditure of much time, labor and patience, are witnessed results unfavorable and discouraging. Good, solid lumps of gold often roll about the cavities that imperfectly inclose them, like loose bullets in flasks, or hang restlessly between teeth whose openings are not sufficiently wide to permit them to escape.

It should be borne in mind that agents of destruction are constantly at work, searching out every defect in our operations, and unless they are so thoroughly performed as to effectually resist the encroachments of these insidious agents, sooner or later our efforts must prove fruitless.

In examining approximal fillings it is not uncommon to find them leaky about their cervical margins, particularly fillings between biscuspids and molars. Careful observers will notice that fillings of this class are, as a rule, less reliable than those elsewhere located. It is true that approximal fillings are more exposed to caries from the difficulty of keeping them free from extraneous substances which find lodgment about the interstices of the teeth, decomposing and generating acids; yet this should admonish us to use extra care and precaution in preparing such cavities. Unfortunately, howevor, they are not usually excavated as much as is needed and so invite the elements of ruin.

Cavities on the bucco-cervical and labial surface of teeth, extending to a line beyond and beneath the margin of the gum, are also liable to similar trouble.

On grinding surfaces of molars and bicuspids, the deep *sulci* between the cusps where the enamel seams are imperfectly united, are points readily invaded by caries. These fissures are not always properly treated. It will not suffice to but partly cut them out, simply forming a small opening in the center of a sulcus, or at a point of junction of two or more seams. Quoting from a distinguished dentist of the present day, " there is altogether too much of this 'botchwork,' this scratching a small hole in the middle of a defective surface, just sufficient to push in a little gold." Every such fissure indicating imperfect fusion of enamel rods, or decalcification of structure, if left undisturbed when preparing the cavity, will present so many vulnerable points for caries to renew their attack. Numberless fillings are thus undermined and destroyed.

It is not always due to an inherent carelessness on the part of the dentist, nor from lack of interest or indifference to duty, that cavities are imperfectly excavated; yet such charges may sometimes be justly preferred; but rather from inability to clearly see and define the exact condition of things.

The secretions from the salivary glands; the exudations from minute follicles that border the necks of the teeth; the flow of blood from the gum when wounded by the instrument; the *debris*, chips, etc., started by the excavator, all tend to fill the cavity and conceal its real condition. How frequently it occurs that after a cavity is supposed to be ready for the filling—having been treated to a good washing and the moisture entirely absorbed—we are surprised to discover that it is not entirely free from caries matter, and more excavating is required.

The truth is, too many of us work in the dark, depending more upon the sense of feeling than of vision; getting but occasional glimpses of some of the cavities we are endeavoring to excavate, just after washing them and mopping out the moisture. Could we but keep the cavities dry, much time and trouble might be saved, and the excavating be more thoroughly done with less pain to the patient; for it is a well-established fact that the dentine is less sensitive to the touch of the instrument when kept free from moisture. After the contents of the cavity are syringed out with warm water and the bulk of decay removed, it is well to apply the rubber dam. Then with a blow-pipe charged with warm air, the cavity may be kept free from chips and dust. Taking all things into consideration, it is no loss of time to make use of the sheet rubber when preparing such cavities as are difficult to keep dry.

The margins of all cavities should be made as smooth as possible. In many cases it is advisable to polish them with sticks of superior stone, also to polish the cavities of incisors with powdered pumice-stone.

For trimming cervical walls, the spoon-shaped excavators are safest and best. These instruments, of various sizes and bent at different angles, do excellent execution, and no operator should be without them. For the approximal margins, in connection with chisels, the sickel-shaped instruments, designed by Dr. Lord, are of great value. To dentists who are accustomed to their use, these instruments are indispensable. The deep fissures on molars and bicuspids are good cases for burring-engines.

If every dentist would take the care and precaution that his operations really require, comparatively few failures would occur ; and in no part of our operations do we need to exercise greater precaution than in preparing cavities for filling, if we desire work that will be enduring and satisfactory to both patient and operator.—*Penn. Journal of Dental Science.*

